# Epidemiology: a partnership of technical expertise and clinical insight

**DOI:** 10.1136/bmjmed-2022-000348

**Published:** 2025-07-01

**Authors:** Stephen Burgess, Deborah A Lawlor

**Affiliations:** 1 MRC Biostatistics Unit, School of Clinical Medicine, University of Cambridge, Cambridge, UK; 2 Cardiovascular Epidemiology Unit, Department of Public Health and Primary Care, University of Cambridge, Cambridge, UK; 3 MRC Integrative Epidemiology Unit at The University of Bristol, University of Bristol, Bristol, UK; 4 North Bristol NHS Trust, Bristol, UK

**Keywords:** Epidemiology, Research design

Like all medical fields, epidemiology is a discipline that can be practiced effectively only in partnership with others. While data analysis may seem like a solitary pursuit, epidemiological datasets are typically built by a group of clinical staff, data collectors, and data managers to identify relevant questions, facilitate access to patients, and provide accurate measurements. Even after study data have been collected and cleaned, epidemiological investigations must rely on knowledge and insight from several domains and individuals with diverse backgrounds to provide meaningful findings: from use of natural science to understand the biological mechanisms to which their findings relate, to social science to understand how the findings can be applied to improve public health or clinical practice. Additionally, practical experience is often required to understand how findings can be implemented. As such, epidemiological investigations are typically beyond the scope of expertise of any single individual (or group from the same discipline).

An effective epidemiologist, either an analyst (who primarily analyses data) or a methodologist (who primarily develops methods for data collection and analysis), requires a skillset incorporating quantitative analysis and critical reasoning skills. However, the application of these skills requires some domain-specific knowledge that often leaves the epidemiologist dependent on others. An analyst may understand what methods can be applied and how to apply them, but the exact specification of the analysis plan requires input from domain-knowledge specialists. Questions such as which clinical or general population should be sampled; how to select the analytical sample; whether to perform age or sex stratified analyses; which subgroup analyses should be attempted; and what variables should be adjusted for, primarily require external knowledge, and methodological ingenuity only secondarily.^1^ More fundamentally, advice is needed to prioritise the important questions to avoid an analysis only becoming an intellectual exercise.

Furthermore, while an epidemiologist may understand what assumptions are required to implement a given method, scientific and clinical insight are required to know whether these assumptions are satisfied or not in practice.^2^ And if they are not satisfied (hint: they never are), to know what extent of deviations from these assumptions is expected. The methodologist has a role in clearly articulating these assumptions and ensuring that the assumptions on which their methods depend are potentially realistic and not mathematically onerous. But judging whether they are reasonable, both in general and in a specific application, relies on input from people with domain knowledge expertise. Quoting Bradford Hill in response to Cochran's seminal paper on causal inference^3^: "What is essential here is a profound knowledge of the field of work under discussion. Merely to go into the origin of a modern epidemic of typhoid fever from a knowledge of statistical records, their strength and weaknesses, will not take one far. One must have a close familiarity with the habits of the bacillus and of the strengths and weaknesses of the laboratory evidence."’

Notable examples show substandard epidemiological work performed by clinicians without the involvement of epidemiologists. The same caution also applies to the interpretation of epidemiological findings by clinicians. A broad example is the conflation of correlation and causation, as seen in a recent editorial misinterpreting a correlation between the timing of coffee drinking and cardiovascular risk as causative.^4^

Collaborative work is best approached from a position of humility and willingness to listen to others. However, academic research poses many challenges to humility, including the strong pressure to publish findings. Good data analysts are in high demand, and their input is often critical to the success of a research project. Research findings are often author-centric, with theories and findings being tied to individuals.^5^ Moreover, the allure of expertise in one domain is enticing to the ego, and often leads to claimed expertise across many domains.^6^ An antidote is scientific humility: working with and listening to others and having the readiness and willingness to admit when initial judgements were wrong. A similar level of openness and accountability is shown by strong clinical teams in their readiness to listen and learn.

How can frontline healthcare professionals and other individuals with medical training work together with analysts and methodologists on epidemiological investigations? Discussion, respect, and humility are required on both sides. Over the past 20 years, a shift has occurred from the prevailing wisdom that reliable causal insights could only come from experimentation (which in epidemiology typically implies a randomised controlled trial) to an acceptance that observational studies also have a key role in our understanding of pathophysiological mechanisms.^7 8^ However, the reliability of such insights is dependent not only on the cleverness of statistical methods but also on assumptions that can never be fully empirically testable, and must rely on external knowledge to justify their validity.

As an example, several within-sibling studies have suggested that conception by assisted reproductive technology markedly reduces the risk of perinatal death. However, clinicians often try a different treatment when an initial one has not been effective, particularly when a critical event has occurred, such as the death of an infant. Clinical colleagues can identify the likely bias mechanism; in this case, the combination of selective fertility (couples are more likely to have a subsequent pregnancy shortly after a perinatal death than couples who have had a live born healthy child) and a change to conception by assisted reproductive technology (a treatment change leading to bias due to carryover where treatment or outcome in one pregnancy affects the treatment or outcome in a subsequent pregnancy). This bias can be counteracted by judicious choice of analytical design and methods. The resulting paper required respectful collaboration between epidemiological analysts and practising clinicians in the field to appropriately identify these biases and propose suitable analytical approaches.^9^

Given emerging trends in large scale data collection and support for the secure use of electronic health records, many epidemiologists will go through their career without ever designing a study or even collecting data. Epidemiologists need respect for those who have collected the data that they analyse. Data cannot properly be analysed without understanding the context in which the data were collected. Engaging with individuals who collect data is essential to correctly interpreting results; including discussions with laboratory workers, study managers, data managers, and frontline clinical staff. Similarly, advice from patient advocacy groups can be critically important in the design and interpretation of analyses to ensure that research questions are relevant to patients and that findings are communicated in a way that patients can understand. Hence, even though epidemiological research can often now be conducted on publicly available data in isolation from applied collaborators, the findings would typically benefit from clinical input.

When working with scientific peers (that is, those with the same background education and training), debates and discussions are often conducted between people on an equal footing. Working with people who have different expertise requires recognising which aspects of an investigation are within our area of expertise, and which require input from others. The importance of diversity in our scientific and social circles should not be underestimated, particularly for ensuring that our research influences and benefits all of society. If our interactions as academic scientists are exclusively with middle class, working age, non-disabled, affluent, public sector workers, then our research will struggle to impact patients outside of those boundaries.

The role of the epidemiologist is not only to analyse but also to investigate, question, object, advocate, propose, articulate, and explain—particularly relating to the assumptions on which the analysis depends. Certainly, some aspects of an investigation are best addressed by the epidemiologist; yet, epidemiologists should not only question aspects of an investigation that are within their area of expertise. Responsibility for a scientific project is shared among all contributors. Good collaboration requires listening critically to others who better understand the scientific and social context of an investigation and seeking out their input in the early stages of development for novel methods and analysis plans. Strong interdisciplinary engagement is essential if we want our research to have both rigour and impact.
